# Breast primary epithelial cells that escape p16-dependent stasis enter a telomere-driven crisis state

**DOI:** 10.1186/s13058-015-0667-z

**Published:** 2016-01-13

**Authors:** Purificación Feijoo, Mariona Terradas, David Soler, Daniel Domínguez, Laura Tusell, Anna Genescà

**Affiliations:** Department of Cell Biology, Physiology and Immunology, Universitat Autònoma de Barcelona, 08193 Bellaterra, Spain

**Keywords:** Mammary epithelial cells, Breast cancer, Telomeres, Chromosome instability

## Abstract

Breast cancer is the most common malignant disease in women, but some basic questions remain in breast cancer biology. To answer these, several cell models were developed. Recently, the use of improved cell-culture conditions has enabled the development of a new primary cell model with certain luminal characteristics. This model is relevant because, after the introduction of a specific set of genetic elements, the transformed cells yielded tumors resembling human adenocarcinomas in mice. The use of improved cell-culture conditions supporting the growth of these breast primary epithelial cells was expected to delay or eliminate stress-induced senescence and lead to the propagation of normal cells. However, no studies have been carried out to investigate these points. Propagation of breast primary epithelial cells was performed in WIT medium on Primaria plates. Immunofluorescence, western blot and qRT-PCR were used to detect molecular markers, and to determine the integrity of DNA damage-response pathways. Promoter methylation of *p16* ^*INK4a*^ was assessed by pyrosequencing. In order to obtain a dynamic picture of chromosome instability over time in culture, we applied FISH methodologies. To better link chromosome instability with excessive telomere attrition, we introduced the telomerase reverse transcriptase human gene using a lentiviral vector. We report here that breast primary epithelial cells propagated *in vitro* with WIT medium on Primaria plates express some luminal characteristics, but not a complete luminal lineage phenotype. They undergo a p16-dependent stress-induced senescence (stasis), and the cells that escape stasis finally enter a crisis state with rampant chromosome instability. Chromosome instability in these cells is driven by excessive telomere attrition, as distributions of chromosomes involved in aberrations correlate with the profiles of telomere signal-free ends. Importantly, ectopic expression of the human *TERT* gene rescued their chromosomal instability phenotype. Essentially, our data show that contrary to what was previously suggested, improved culture conditions to propagate *in vitro* mammary epithelial cells with some luminal characteristics do not prevent stress-induced senescence. This barrier is overcome by spontaneous methylation of the *p16* ^*INK4a*^ promoter, allowing the proliferation of cells with telomere dysfunction and ensuing chromosome instability.

**Electronic supplementary material:**

The online version of this article (doi:10.1186/s13058-015-0667-z) contains supplementary material, which is available to authorized users.

## Background

Breast carcinomas exhibit a great diversity in clinical parameters. The tumor cell phenotype can be influenced by different factors, including genetic and epigenetic changes and cell-stroma interactions, but also by the initial normal epithelial cell type that serves as a precursor of the tumorigenic cells [1]. The normal human mammary epithelium consists basically of an inner, luminal layer of milk-producing cells and an outer, myoepithelial layer of cells that contract to bring the milk to the nipple. The majority of breast cancers are diagnosed in women older than 50 years, and belong to luminal subtypes [2–4]. Molecular studies of normal breast tissue sections have revealed an age-related decrease in the proportions of myoepithelial cells that could be linked to breast cancer progression, as this particular cell lineage is responsible for maintaining cell polarity [5, 6]. In contrast, breast cells propagated in vitro under standard conditions suffer an opposite trend, with progressive loss of cells of the luminal lineage. The standard in vitro culture of breast organoids with mammary epithelial growth medium (MEGM), mammary epithelial growth medium-basal (MEpiCM) or mammary epithelial growth medium MCDB-170 allows growth of cells that after a few population doublings, express mostly myoepithelial molecular markers. These cells propagated in vitro are termed human mammary epithelial cells (HMECs) [7, 8]. Long-term maintenance of the luminal phenotype in culture has been a challenge for a long time. In 2007, the laboratory of Weinberg developed a new culture medium termed WIT, which along with the use of a modified plastic surface (Primaria plates), allowed the propagation of breast epithelial cells with some luminal characteristics, such as the presence of Claudin-4 and absence of CD-10 [1]. These in vitro propagated cells were named breast primary epithelial cells (BPECs). Most importantly, HMECs and BPECs derived from the same healthy donors generated distinct types of tumors in immunocompromised mice when experimentally transformed with the same set of genetic elements. While the introduction of *H-RAS, hTERT* and *SV40 LT/st* in HMECs yielded tumors similar to squamous cell carcinomas, transformed BPECs were highly tumorigenic and metastatic, and yielded tumors closely similar to human breast adenocarcinomas [1], which is the most common type of breast cancer in women.

While normal human epithelial cells retain a stable genotype, carcinomas usually express genomic instability, which accelerates the accumulation of mutations that drive tumor genesis. The most common form of genomic instability in human cancers, including breast carcinomas, is chromosomal instability (CIN), which consists of a high rate of changes in number and structure of chromosomes over time. The molecular basis of CIN is beginning to be explored. CIN can result from oncogene-induced DNA replication stress, such as that imposed by mutated *RAS*, which produces a persistent mitogenic stimulation resulting in an increased number of active DNA replication origins, collapsed replication forks, and DNA damage [9, 10]. Besides oncogene-induced replication stress, telomere dysfunction has been discovered as a major source of tumor-associated CIN: unrestrained proliferation of cells with disabled cell-cycle checkpoints results in loss of protective telomere function with induction of a DNA-damage response and karyotype instability [11]. Thus telomeres must be added to the list of critical caretakers responsible for maintaining genome integrity.

Efforts to develop models of early carcinogenesis in non-transformed human cells are essential to investigate the mechanisms underlying chromosome instability because studies performed in tumor-derived cell lines may not be reflective of the initiation of genomic instability process. In this line, human mammary epithelial cell models such as HMECs have provided valuable clues about the origin of CIN in the myoepithelial lineage of mammary cells. HMECs undergo stress-associated growth arrest within a few weeks of in vitro propagation [12, 13] that is associated with increased expression of the tumor suppressor p16^INK4a^. This stress-associated barrier - known as stasis - can be bypassed in HMECs propagated in vitro by spontaneous promoter methylation of the *p16* ^*INK4a*^ gene [14]. Others, and ourselves, reported that HMECs with silenced *p16* ^*INK4a*^ display critical telomere erosion that fuels chromosome instability in all its manifestations, i.e., chromosome rearrangements [14, 15], segmental duplications [16], aneuploid chromosome segregations [17, 18], and polyploidization [14, 19].

Less is known about BPECs, of which their relevance as a cellular model in human breast carcinogenesis is unquestionable. It was reported that in contrast to mammary epithelial cells cultured in standard conditions, the p16^INK4a^ protein was not significantly induced in cells cultured in WIT medium on Primaria plates. It was suggested that these improved culture conditions allowed the unimpeded long-term propagation of a population of mammary epithelial cells among which senescence is delayed or eliminated [1, 20]. We aimed to investigate whether BPECs, which, are able to produce adenocarcinoma-like tumors after experimental transformation, develop mechanisms to bypass senescence with ensuing telomere dysfunction and chromosome instability.

## Methods

### Cells and culture conditions

BPECs and HMECs were obtained from mammoplasty specimens of disease-free patients, and were propagated according to conditions described by Ince and colleagues [1]. All necessary ethical approvals and consents were obtained for the collection and use of tissue samples for research purposes. BPECs were cultured in BD Primaria surface (BD Bioscience) using WIT-P-NC medium (initially supplied by Stemgent, Cambridge, MA, USA, ref 00–0051, and more recently by Cellaria, Boston, MA, USA, ref CM-0104) supplemented with 100 ng/ml cholera toxin (Sigma-Aldrich, Tres Cantos, Spain, ref C8052). HMECs were cultured in standard plates with serum-free MEpiCM (ScienCell, Research Laboratories, Carlsbad, CA, USA). BPECs and HMECs were grown at 37 °C and in 5 % CO_2_. The number of accumulated population doublings (PDs) per passage was determined using the equation:

$$ \mathrm{P}\mathrm{D}\kern0.5em =\kern0.5em {\mathrm{PD}}_{\mathrm{initial}}+\kern0.5em  \log \kern0.5em \left(N\kern0.5em \mathrm{viable}\ \mathrm{cells}\ \mathrm{harvested}/N\kern0.5em \mathrm{viable}\ \mathrm{cells}\ \mathrm{plated}\right)/ \log\ 2. $$

BPECs expressing *hTERT* (04BPEC-hTERT, 05BPEC-hTERT and 12BPEC-hTERT) were generated by lentiviral transduction with the *hTERT* gene at a pre-stasis PD (04BPECs at PD 3, 05BPECs at PD 4 and 12BPECs at PD 10) in the presence of 4 mg/ml Polybrene (Sigma-Aldrich). For propagation of BPEC-hTERT cell lines, WIT-T-NC medium (Cellaria, ref C10103) was supplemented with 25 ng/ml cholera toxin (Sigma-Aldrich, ref C8052).

### Immunodetection of protein markers

For immunofluorescent detection of protein markers under a fluorescence microscope, cells were plated into chamber slides, fixed with paraformaldehyde 4 % for 10 minutes permeabilized for 15 minutes in 1 × PBS 0.5 % Triton X100 solution, rinsed twice with 1 × PBS and, blocked in PBS 0.1 % Tween20 2 % fetal calf serum for 1 h at room temperature. Antibodies used were rabbit anti-Claudin-4 (Abcam, Cambridge, UK, Ref 1504), mouse anti-CD-10 (Abcam, Ref. 10323), rabbit anti-cytokeratin K-14 (Covance, Madrid, Spain, Cat# PRB-155P-100) and rat anti-cytokeratin K-19 (Troma III, Iowa, USA) and rabbit anti-human TERT (Rockland, Limerick, PA, USA). Staining for antibodies against Claudin-4, CD-10, cytokeratin K14 and cytokeratin K19 was evaluated under an optical epifluorescence microscope with specific filters for each of the fluorochromes used, and images were obtained using Isis Fluorescence Imaging software (MetaSystems GmbH, Altussheim, Germany). Mouse anti-CD-10 (BioLegend, San Diego, CA, USA) and mouse anti-CD-227 (BD Pharmigen, Franklin Lakes, New Jersey, USA) were detected by flow cytometry.

### Western blot analysis

Proteins were extracted with CHAPS lysis buffer, quantified with NanoDrop 2000 (Thermo Fisher Scientific, Barcelona, Spain), denatured at 70 °C, separated on a 10 % SDS-PAGE gel (Novex) and transferred on a nitrocellulose membrane. Antibodies used were rabbit anti-Claudin-4 (1:1000, Abcam, Ref 1504), mouse anti-p16^INK4a^ (1:1000, Neomarkers, Freemont, CA, USA), mouse anti-p53 (1:1000, Santa Cruz Biotechnologies, Heidelberg, Germany), rabbit anti-phospho S15 p53 (1:1000, Invitrogen) and mouse glyceraldehyde-3-phosphate dehydrogenase (GAPDH) (1:1000, Abcam) diluted on 1 × PBS-3 % BSA 0.1 % Tween20. Anti-mouse and anti-rabbit horseradish peroxidase (HRP) conjugate was used as secondary antibody (1:2000, Millipore, Madrid, Spain). Chemiluminescent detection of antibodies was performed using HRP solution and luminol (Immobilion Western kit, Millipore).

### RNA collection and quantitative reverse transcription PCR

Total RNA was isolated from cells using Trizol reagent (Ambion, Fisher Scientific, Pittsburgh, PA, USA). After chloroform addition and centrifugation, an aqueous phase containing RNA was obtained. RNA was purified and treated with DNase using the Maxwell RSC simply RNA Cells Kit (Promega, Madrid, Spain). RNA was reverse transcribed into cDNA using iScript (BioRad, Madrid, Spain). Quantitative PCR (qRT-PCR) was performed with SYBR green (BioRad) using CFX96 thermal cycler (BioRad). Primers used for qRT-PCR are listed in Additional file 1: Table S1. Primers were designed using Primer3 online software. GAPDH RNA levels were used for normalization.

### Pyrosequencing methylation analyses

Genomic DNA extraction was performed with the Gentra Puregen Kit (Qiagen): 350 ng of DNA per sample were treated with bisulphite using the EZ DNA Methylation-DirectTM kit (Zymo Research Corporation, Irvine, CA, USA). The promoter methylation status of *p16* ^*INKa4*^ was analyzed using the PyroMark CpG Assay PM00039907 (Qiagen), which allows the analysis of six CpG sites of the *P16* ^*INK4a*^ promoter by pyrosequencing. First, bisulphite-converted DNA was amplified by PCR using the target-specific forward and reverse primers (one of which is biotin 5’-labeled) provided with the kit and with AmpliTaq Gold DNA Polymerase (Applied Biosystems, Madrid, Spain). After checking PCR products by agarose gel, biotinylated amplicons were conjugated with streptavidin and recovered using the PyroMark Vacuum Prep Workstation (Qiagen). Pyrosequencing was carried out on a PSQ96HS Pyrosequencing instrument (Biotage, Madrid, Spain) using the target-specific sequencing primer provided with the kit. Finally, the analysis was performed using the Pyro Q-CpG Software (Qiagen, Hilden, Germany). Methylation status of the *p16* ^*INK4a*^ promoter was quantified in terms of methylation mean, which is the mean percentage of methylated cytosines in five CpG sites. In order to avoid technical differences, duplicates were performed on each sample.

### Abnormal nuclear morphologies assay

To obtain binucleated cells, cytokinesis was blocked by adding cytochalasin B (Sigma-Aldrich) at a final concentration of 6 μg/ml [21]. Binucleated BPECs were stained with propidium iodide and 4',6-diamidino-2-phenylindole (DAPI) to observe the two nuclei included in the same cytoplasm, and limit the scoring of nucleoplasmic bridges, micronuclei and nuclear buds to the cells that have divided once. Scoring was done following standard previously established criteria [22].

### Metaphase cell preparations for cytogenetic analyses

Exponentially growing BPECs were treated with Colcemid 0.02 μg/ml for 4 h, followed by hypotonic shock and methanol/acetic fixation. Cell suspensions were dropped onto slides and mounted with DAPI staining. Metaphase karyotyping was performed by reverse DAPI staining, which results in a reproducible G-band-like pattern that allows chromosome identification.

### Fluorescence *in situ* hybridization (FISH)

For centromere and telomere peptide nucleic acid (PNA)-FISH slides containing metaphase spreads were treated with pepsin/HCl, rinsed in PBS and post-fixed with formaldehyde-MgCl_2_. The hybridization mix contained a pancentromeric PNA-fluorescein isothiocyanate (FITC) (FITC-AAACACTCTTTTTGT-AGA) (Panagene, Daejeon, Korea) and a pantelomeric PNA-Cy3 (CCCTAA) probe (PE Biosystems, Foster City, CA, USA). DNA on slides was denatured at 80 °C for 90 seconds. Hybridization was at room temperature for 2 h, and post-hybridization washing steps were carried out with 70 % formamide and Tris-NaCl-Tween 20 buffer. Finally, slides were dehydrated and counterstained with DAPI. Fluorescence signals were visualized under an Olympus BX microscope equipped with epifluorescent optics specific for each fluorochrome. Images were captured and analyzed using Cytovision software (Applied Imaging, San Jose, CA, USA). We identified the set of telomere signal-free ends in each donor sample by scoring the frequency of unlabeled chromosome ends. The length of the telomeric sequences in chromosome arms with undetectable signals using adequate fluorescence microscope filters must be shorter than 0.5 kb of TTAGGG repeats, which is the resolution of PNA-FISH telomeric probes on metaphase chromosomes [23].

M-FISH was performed on metaphase spreads of 12BPEC at PD 44 and 14BPEC at PD 18 and 30. Slides were treated with pepsin/HCl and post-fixed with formaldehyde-MgCl_2._ Hybridization probe M-FISH (Vysis, Abbot Molecular, Illinois, USA) was added to the slides denatured at 80 °C for 90 seconds. Hybridization was performed overnight, and post-hybridization washing steps were carried out with 0.4 × SSC at 73 °C, and with 2 × SSCT (Sodium Saline Citrate) at room temperature. Finally, slides were dehydrated and counterstained with DAPI. Signals were visualized under a microscope equipped with epifluorescent optics (Olympus BX61 epifluorescence microscope), a CCD camera, specific filters (DAPI, Far red, Cy3,Aqua, FITC and Gold) and were captured and analyzed using Cytovision software (Applied Imaging, San Jose, CA, USA).

For chromosome-specific centromeric FISH, slides were permeabilized with 4 % formaldehyde and dehydrated. A sample DNA denaturing step was carried out using 2 x SSC -70 % formamide for 10 minutes at 73 °C. After a cold ethanol serial dehydration, samples were incubated with a mixture of three probes for chromosomes 1, 4 and 18 (Abbott, Abbot Park, IL, USA) or for chromosomes 6, 12 and 17 (OligoFISH Probes, Cellay Inc, Cambridge, MA, USA). Before hybridization, the slides were pre-treated with pepsin (0.1 mg/ml; Sigma-Aldrich) in 10 mM HCl and post-fixed in 37 % formaldehyde in PBS/1 M MgCl_2_. All probes were applied according to the manufacturer’s instructions. Finally, slides were dehydrated and mounted in anti-fade solution containing DAPI. Fluorescence signals were visualized under an Olympus BX microscope equipped with epifluorescent optics specific for each fluorochrome.

### Statistical analysis

All of the frequencies were calculated based on events that occurred. Data analysis was carried out with the statistical program SPSS 15.0 for Windows (SPSS Inc, University of Chicago, USA). Statistical differences between the analyzed samples were considered significant with a *P* value <0.05.

## Results

### Breast epithelial cells propagated in WIT medium are not fully luminal lineage cells

**Fig. 1:**
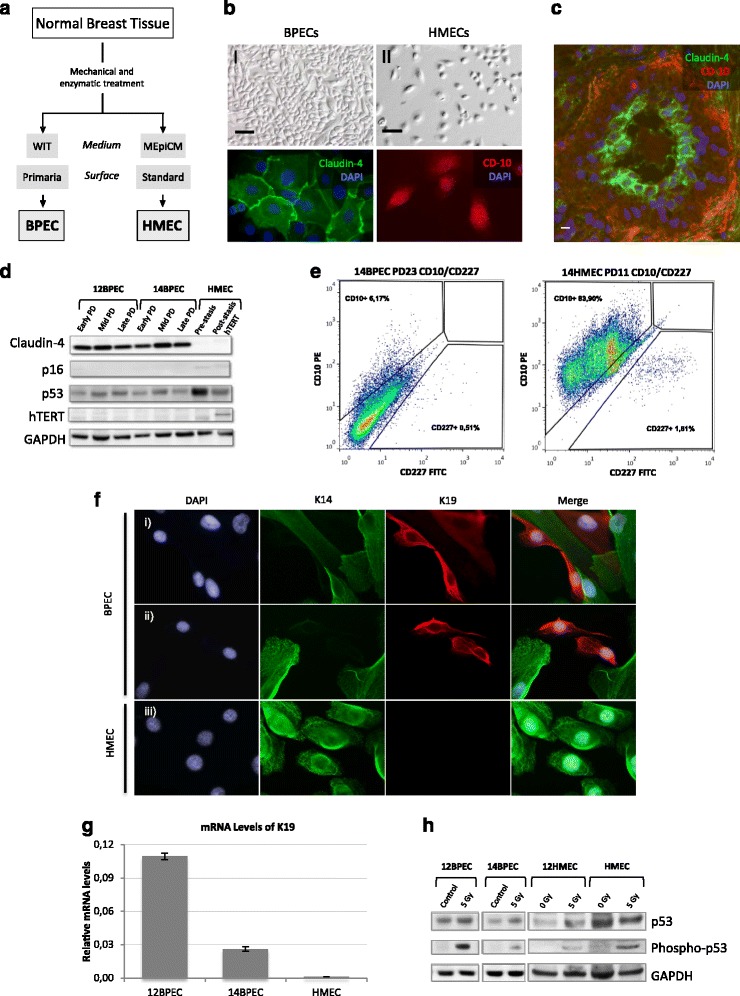
Characterization of human mammary epithelial cells. **a** Schematic representation of steps for the derivation of two human mammary epithelial cell types, breast primary epithelial cells (*BPECs*) and human mammary epithelial cells (*HMECs*). **b** Images of BPECs and HMECs showing: **I** BPECs that have been grown in WIT medium and on tissue culture plates with a modified attachment surface (Primaria); **II** HMEC that have been grown in mammary epithelial growth medium-basal (MEpiCM) and on regular culture plates; **III** and **IV** detection of luminal-specific Claudin-4 (*green*) and myoepithelial specific CD-10 (*red*) proteins in BPECs and HMECs by immunofluorescence. **c** Paraffin section of a breast sample after immunofluorescent detection of Claudin-4 (*green*) and CD-10 (*red*). **d** Western blot for detection of Claudin-4, p16^INK4a^, basal p53 and human telomerase reverse transcriptase (*hTERT*) in BPECs at an early, a mid and a late population doubling (PD) and in HMECs at pre-stasis and post-stasis; glyceraldehyde-3-phosphate dehydrogenase (*GAPDH*) was used as loading control. **e** Sorting of BPEC and HMEC cultures for CD-227 and CD-10. **f** Immunofluorescent detection of keratins 14 (*K14*) and 19 (*K19*) on BPEC and HMEC cultures. In the BPEC cultures, we found cells positive for K14 and positive for K19 (**i** and **ii**). But in the HMEC culture (**iii**), we only observed K14-positive cells. **g** mRNA levels of K19 in 12BPECs, 14BPECs and HMECs, as obtained by qRT-PCR. **h** Western blot for detection of basal p53 and the s15 phosphorylated form of p53 in irradiated and non-irradiated BPECs. *DAPI* 4',6-diamidino-2-phenylindole

Cells for molecular and cytogenetic analyses were obtained from normal disease-free breast tissue collected from two surgical mammoplasties (donors number 12 and 14). Isolation of BPECs from the original breast tissue and their propagation in vitro using WIT medium and Primaria plates was performed strictly following the experimental procedures described by the Weinberg laboratory in their original manuscript [1] (Fig. 1a). The two specimens obtained are hereinafter referred to as 12BPEC and 14BPEC, and were systematically analyzed at three different PDs during their culture in vitro: early (PD6 for both cell lines), mid (PD25 and PD18 for 12BPEC and 14BPEC, respectively) and late (PD44 and PD30, respectively). Isogenic HMECs were obtained by culture of organoids from donors number 12 and 14 on standard MEpiCM medium and a plastic surface, and are referred to as 12HMEC and 14HMEC.

Both HMECs and BPECs had typical epithelial morphology (Fig. 1b). According to the results reported by Ince et al. [1], our BPEC cultures expressed Claudin-4, a protein that is exclusive of the inner luminal cell layer of the normal breast epithelium (Fig. 1c), at the three analyzed PDs (Fig. 1b and d). In addition, CD-10, which is exclusively expressed in the outer myoepithelial cell layer (Fig. 1c), was highly expressed in HMECs, but not in BPEC cultures (Fig. 1b and e). Moreover, neither of the two cell populations showed protein labeling of the luminal marker CD-227 (Fig. 1e). In this context, it has been described that some markers as CD-227 are lost during the tissue dissociation protocol [24, 25]. We also analyzed protein expression of cytokeratin K14, a myoepithelial marker, and K19, a luminal marker, which were not checked by Ince et al. [1]. We observed cells with one or the other, or even both, molecular markers in BPEC cultures, whereas cell HMEC cultures only expressed K14 (Fig. 1f). Moreover, K19 mRNA levels were higher on BPECs than on HMECs (Fig. 1g). Therefore, in agreement with Ince et al. [1], BPECs did not show a protein expression program characteristic of fully differentiated luminal cells, but were considerably less myoepithelial than HMECs.

The cell lines were primary, and in accordance with this, western blot confirmed that levels of hTERT were undetectable in BPEC lines and in isogenic HMECs (Fig. 1d). Regarding the pathways of DNA damage response, basal levels of p53 were detected in both cell lines by western blot (Fig. 1d, h). Most importantly, increased levels of p53 phosphorylated at serine 15 were detected in BPECs after radiation exposure (Fig. 1h), suggesting the integrity of the p53 response.

### Breast primary epithelial cells undergo p16-dependent stasis

**Fig. 2:**
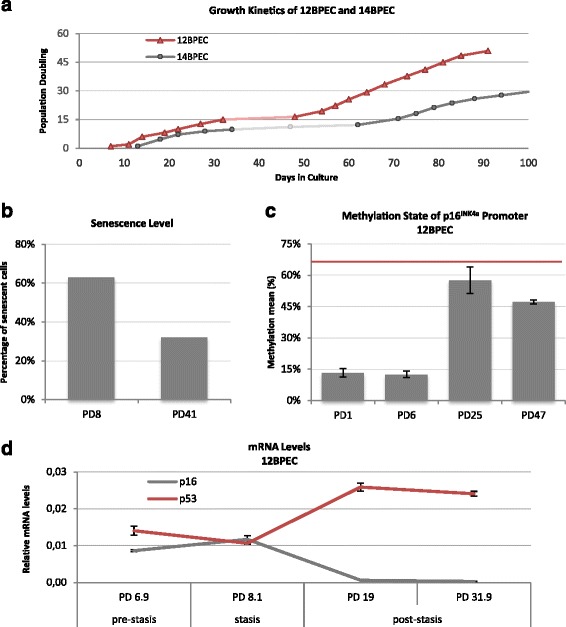
Propagation of human mammary epithelial cells. **a** Growth kinetics of 12-breast primary epithelial cells (*12BPEC*) and 14BPEC (population doubling (*PD*) vs days in culture). **b** Frequencies of 12BPEC at PD8 and PD41 expressing senescence-associated β-galactosidase marker. **c** Methylation mean of the *p16* ^*INK4a*^ gene promoter in 12BPECs at different population doublings, as obtained by pyrosequencing methylation analyses. The red horizontal line represents the frequency of *p16* ^*INK4a*^ promoter methylation in post-M0 human mammary epithelial cells. **d** Expression profile of p16 and p53 in 12BPEC at pre-stasis, stasis and post-stasis population doublings. Expression was tested using qRT-PCR, and data were normalized using glyceraldehyde-3-phosphate dehydrogenase (GAPDH) RNA levels

Typically, HMECs go into a stress-induced senescence, called stasis, a few weeks after the cells are explanted from breast organoids. Some HMECs overcome this barrier and continue proliferating by the methylation-mediated silencing *p16* ^*INK4a*^ gene [14]. In contrast to standard medium, in which HMECs are propagated, WIT medium and improved culture conditions were suggested to eliminate the induction of stress-related senescence allowing the cells to proliferate unimpeded in culture up the point of crisis [1, 20]. However, we recorded PDs versus time in BPECs and observed a plateau compatible with stasis in both BPEC lines (Fig. 2a). In concordance with stress-induced senescence, we observed a sharp increase in the expression of β-galactosidase (SA-βGal) in BPEC 3 weeks after the cells were explanted from the breast organoids, followed by a decrease at later PD (63 % positive staining for SA-βGal in 12BPEC at PD8 and 32 % at PD41) (Fig. 2b). Thus, in contrast to what was asserted previously, propagation of breast primary epithelial cells in WIT medium does not prevent stress-induced senescence.

12BPEC and 14BPEC did not show p16^INK4a^ protein expression at any of the PD analyzed (Fig. 1d). However, lack of detectable p16^INK4a^ protein in BPECs does not necessarily mean that the cells are unstressed. In this context, it has been established that HMECs might overcome stasis by spontaneous silencing of the p16^INK4a^ tumor suppressor gene. To investigate whether BPECs were suffering a similar phenomenon as described for HMECs, promoter methylation of *p16* ^*INK4a*^ was assessed quantitatively by pyrosequencing. We observed a varying methylation level of the p16^INK4a^ promoter throughout the BPEC culture (Fig. 2c and Additional file 2: Figure S1a). At the early PDs, p16^INK4a^ promoter methylation level was low (13.3 % in 12BPEC and 12.0 % in 14BPEC), while it increased at mid and late PDs (57.6 % at PD25 and 47.2 % at PD47 in 12BPEC; 26.8 % at PD18, 30.3 % at PD25, and 29.73 % at PD30 in 14BPEC). These methylation levels in mid and late PDs of BPECs were similar to those observed in commercial post-stasis HMECs (53 % in 1001 HMECs). Accordingly, p16 mRNA levels decreased over time in culture, while p53 levels increased (Fig. 2d). In conclusion, similar to HMECs, breast epithelial cells propagated in WIT medium and Primaria surface suffer a p16-dependent stress-induced senescence, which is overcome by spontaneous methylation of the p16^INK4a^ promoter.

### BPECs show abnormal nuclear morphologies and chromosome instability

**Fig. 3:**
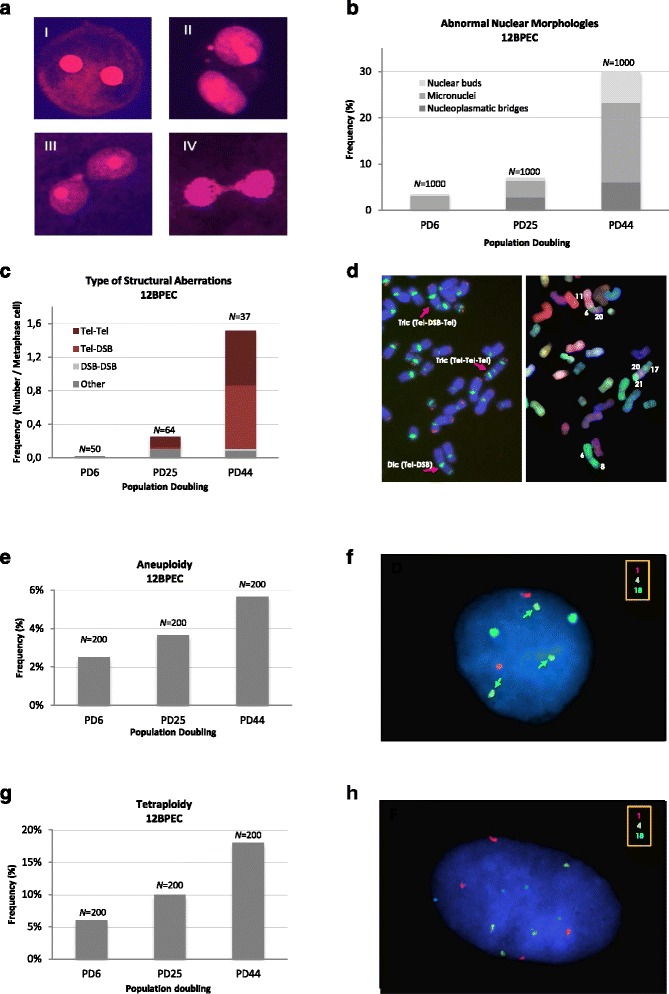
Breast primary epithelial cells (*BPECs*) show chromosome instability. **a** Binucleated BPECs stained with 4',6-diamidino-2-phenylindole (DAPI) showing: **I** a normal binucleated cell; **II** a binucleated cell with two micronuclei, **III** with a nuclear bud, and **IV** with a nucleoplasmic bridge. **b** Frequency of abnormal nuclear morphologies per binucleated cell in 12BPECs at initial, medium and late population doubling (PD). *Bar* 10 μm. **c** Frequencies of the different types of structural chromosome aberrations observed in metaphase plates of 12BPEC at an early, a mid and a late PD.  **d** Representative examples of structural chromosome aberrations as detected by centromere + telomere peptide nucleic acid (PNA)- fluorescence *in situ* hybridization (FISH) (*left*) and M-FISH (*right*) probes. **e** Frequencies of aneuploidy (showing results for 12BPECs at an early, a mid and a late PD). Each column represents accumulated aneuploidy frequencies from the six analyzed chromosomes (1, 4, 6, 12, 17, 18). **f** Representative image of single nucleus hybridized with centromeric probes specific for chromosome 1, 14 and 18. *Arrows* three copies of chromosome 18. **g** Frequencies of tetraploidy. Each *bar* represents the frequency of cells with a tetraploid set of chromosomes as ascertained after hybridization with probes for chromosomes 1, 4, 6, 12, 17 and 18 (shown results for 12BPECs at an early, a mid and a late PD). **h** Representative image of a tetraploid nucleus

In spite of the importance of BPECs as a cellular model for breast cancer research, no studies have been carried out on their genome integrity. The detection of binucleated cells in our BPEC cultures led us to suspect that the cells presented with genomic aberrations. Thus, we examined abnormal nuclear morphologies (ANMs), often used as indicators of chromosomal instability, in 12BPEC (Fig. 3a and b) and 14BPEC (Additional file 3: Figure S2a) at three different PDs. Specifically, we analyzed the presence of micronuclei, nuclear buds, and chromatin bridges. The frequency of cells with ANM increased dramatically from early to late PDs in 12BPEC (3.5 % of cells showing ANM at the early, 7.1 % at the mid, and 30.0 % at the late PD analyzed), and in 14BPEC (2.8 % at the early, 11.0 % at the mid, and 26.2 % at the late PD analyzed), thus revealing that BPECs propagated in vitro are unable to maintain the integrity of their genome.

In order to obtain a dynamic picture of structural chromosome instability in BPECs throughout the time in culture, we applied M-FISH, telomeric, and centromeric hybridization, and cytogenetic analysis by DAPI reverse banding. Structural chromosome abnormalities increased in 12BPEC and 14BPEC with PDs (Fig. 3c and Additional file 3: Figure S2b). Both cell lines had a normal diploid karyotype at the earliest PD, with frequencies of chromosome aberrations as low as 0.02 in 12BPEC and 0.06 in 14BPEC. At the mid PD analyzed, we observed an increase in the frequency of chromosome rearrangements, which preferentially affected the ends of the chromosomes (0.25 structural chromosome aberrations per cell in 12BPEC and 0.78 in 14BPEC). At the latest PD analyzed, the frequency of rearrangements increased dramatically (1.5 structural chromosome aberrations per cell in 12BPEC and 1.11 in 14BPEC) and involved not only the ends, but also internal loci in the chromosomes (Fig. 3c and d). Thus, chromosome reorganizations gained complexity throughout the culture, pointing to a progressive scrambling of the genome in BPECs.

Fluorescence *in situ* hybridization with centromeric specific DNA probes was applied to analyze changes in ploidy. We used probes for six chromosomes, and observed that the frequency of cells with abnormal chromosome numbers increased progressively through the in vitro cell culture (Fig. 3e, f; 2.5 % at PD6, 3.7 % at PD25 and 5.7 % at PD44 in 12BPEC, X^2^ *P* <0.05; results for 14BPEC shown in Additional file 3: Figure S2c). Additionally, the frequency of tetrapolyploid cells (with four centromeric signals of each chromosome analyzed) increased with the PDs and was three times higher in the late PD than in the early one (Fig. 3g, h; 6 % at PD6 vs 18 % at PD44 in 12BPEC; results for 14BPEC shown in Additional file 3: Figure S2d). Thus, we conclude that BPECs grown in WIT are not able to maintain the integrity of their genomes, and they present structural rearrangements and abnormalities in the segregation of whole chromosomes and in cell ploidy.

### Distributions of chromosomes involved in rearrangements correlate with the profiles of telomere signal-free ends

**Fig. 4:**
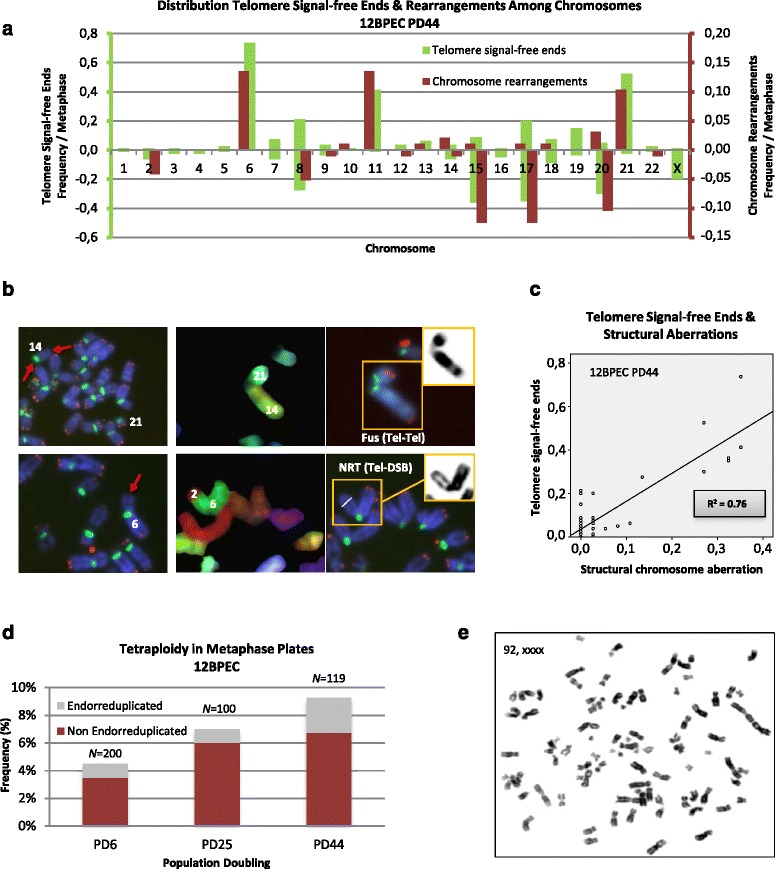
Chromosomal instability in breast primary epithelial cells (*BPECs*) correlates with telomere erosion. **a** The distribution of individual chromosomes involved in rearrangements correlates with the profile of individual chromosomes with telomere signal-free ends (*bar diagram* corresponds to 12BPEC at PD44). **b** Partial metaphases showing two representative examples (one in each file) of individual chromosomes with telomere signal-free ends (*left row*) and the involvement of these particular chromosome arms in rearrangements (M-fluorescence *in situ* hybridization (FISH) in the middle row and telomere + centromere FISH in the *right row*). **c** Regression between the frequencies of telomere signal-free ends among the different chromosomes and their involvement in rearrangements (*diagram* corresponds to 12BPEC at PD44). **d** Frequencies of tetraploid 12BPEC at metaphase (endo-reduplicated and non-endo-reduplicated) at the three population doublings (PDs) analyzed, with 100 metaphase plates analyzed for each donor and PD. **e** Representative image of a non-endo-reduplicated tetraploid BPEC at metaphase

Multiple lines of evidence indicate that telomeres protecting the ends of chromosomes are centrally involved in the maintenance of genome integrity, preventing the formation of unstable dicentric chromosomes [26]. However, telomere dysfunction has not been reported before in BPECs. To explore whether there is a connection between telomere function and the observed CIN in this lineage of mammary epithelial cells, we examined metaphase cells for the presence of chromosome ends lacking telomere hybridization signals. Progressive telomere attrition was observed as 12BPEC and 14BPEC proliferated in vitro (results shown in Additional file 4: Figure S3a, b). In accordance with a telomere basis for the CIN observed in BPECs, structural chromosome aberrations preferentially involved the chromosome arms that most frequently lacked visible telomere signals (Fig. 4a). For instance, chromosome arms displaying the highest frequencies of telomere signal-free ends at the latest PD analyzed in 12BPECs are 6p, 8q, 11p, 15q, 17q, 20q and 21p (Fig. 4a, b). These particular chromosome arms are also more frequently involved in chromosome aberrations at this PD than other ends (30.4 % of structural chromosome aberrations involved chromosome arm 11p, 28.6 % 6p, and 26.8 % 17q) (*R* ^2^ 0.76; Fig. 4c). In addition, a progressive increase in the number of non-endo-reduplicated tetraploid cells, presumably originating from mitotic failure, was observed along with PDs (Fig. 4d, e; Additional file 5: Figure S4). These results are consistent with an increase in chromosome aberrations arising from telomere dysfunction in BPECs.

### Ectopic expression of *hTERT* rescues *c*hromosomal instability phenotype of BPECs

**Fig. 5:**
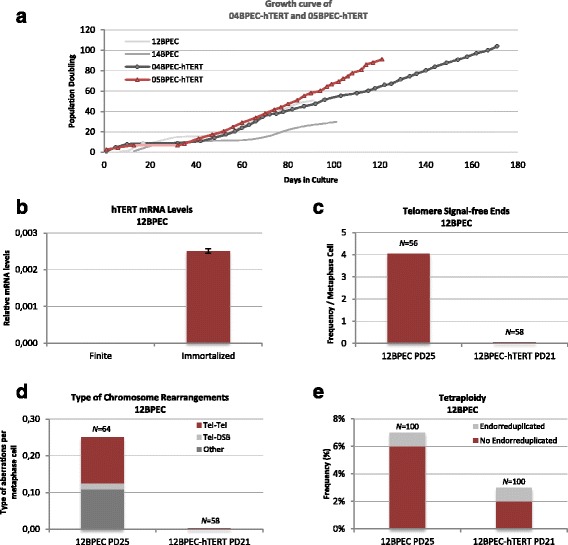
Ectopic expression of human telomerase reverse transcriptase (*hTERT*) rescues chromosomal instability phenotype of breast primary epithelial cells (*BPECs*). **a** Growth kinetics of 04BPEC-hTERT and 05BPEC-hTERT. **b** mRNA levels of hTERT assessed by qRT-PCR in 12BPEC-hTERT versus finite (non-tranduced) BPECs. **c** Frequencies of chromosomes with telomere signal-free ends are dramatically reduced after transduction with *hTERT* (12BPEC at PD25 and 12BPEC-hTERT at PD21). **d** Reduced frequencies of structural chromosome rearrangements after transduction with *hTERT* (12BPEC at PD25 and 12BPEC-hTERT at PD21). **e** Frequencies of tetraploid nuclei are reduced after transduction with *hTERT* (12BPEC at PD25 and 12BPEC-hTERT at PD21)

To better link CIN in BPECs with excessive telomere attrition, using a lentiviral vector we introduced the telomerase reverse transcriptase human gene (*hTERT*) in primary cultures of BPECs from four different donors (12BPEC, 14BPEC, 04BPEC and 05BPEC). In all cases infection was carried out in pre-stasis cells. Only three of the cell lines could be properly transduced (12BPEC-hTERT, 04BPEC-hTERT and 05BPEC-hTERT). Expression of hTERT in the transduced cells was confirmed by qRT-PCR in the three cell lines (Fig. 5b and Additional file 6: Figure S5a, b). Growth kinetics of 04BPEC-hTERT and 05BPEC-hTERT (Fig. 5a) show that the introduction of *hTERT* gene does not prevent stasis, but has an impact on the replicative lifespan of the culture, which become immortal. Indeed, the methylation state of the *p16* ^*INK4a*^ promoter of 05BPEC-hTERT cells increased from 11.8 % to 50.9 % after stasis, reaching similar levels to 12BPEC and 14BPEC at post-stasis. These results indicate that immortal BPECs inactivate *p16* ^*INK4a*^ gene during stasis.

Regarding the impact of *TERT* transduction in telomere length, we observed a significant decrease in the frequency of telomere signal-free chromosome ends in the three transduced cell lines (for instance, 4.05 telomere signal-free ends per metaphase in non-transduced 12BPECs at PD25 vs no telomere signal-free ends in 12BPEC-hTERT at PD21; Fig. 5c). In accordance with a telomere-dependent origin of CIN in BPECs, restoration of telomerase activity was translated into a dramatic decrease of chromosome aberrations. In 12BPEC-hTERT at PD21, no structural chromosome aberrations were observed (Fig. 5d), in contrast to the 0.25 chromosome rearrangements per cell reported in isogenic 12BPEC at PD25. In addition, the frequency of non-endo-reduplicated tetraploid cells, presumably originated by mitotic failure, was 6 % in non-transduced cells and decreased to 2 % in 12BPEC-hTERT (Fig. 5e). Similar results were observed in the other two transduced cell lines, 04BPEC-hTERT and 05BPEC-hTERT, as compared with the isogenic primary precursors (Additional file 7: Table S2). Together, these results reveal that restoration of telomerase before stasis reduces structural and ploidy aberrations, and demonstrate that short dysfunctional telomeres in BPECs cause the observed chromosome instability in the primary cell lines.

## Discussion

We obtained breast primary epithelial cells expressing some luminal molecular markers and examined whether they developed telomere-based chromosome instability when propagated in vitro. Two major findings were obtained:

1. Spontaneous methylation of the *p16* ^*INK4a*^ promoter region is observed in BPECs cultured in WIT medium. The silencing of this gene allows BPEC propagation beyond stress-induced senescence.
2. Finite lifespan BPECs develop telomere dysfunction and subsequent telomere-based chromosome instability.

Previous studies in finite lifespan HMECs propagated in vitro under standard conditions have shown that they encounter a first barrier to indefinite proliferation associated with stress in culture [12, 13]. This stress-associated barrier can be overcome in cultured HMECs by multiple types of alterations in the RB pathway, most commonly by spontaneous epigenetic silencing of the gene encoding p16^INK4a^ [27]. Improved cell culture conditions recently developed to allow the proliferation of BPECs were suggested to prevent the over-expression of p16^INK4a^ and the stress-induced senescence [1, 20]. In accordance with the results reported by Ince et al. [1], we did not detect p16 protein, but analysis of mRNA expression in cells near stasis showed that the p16 mRNA levels increased at stasis and decreased in post-stasis BPECs. In this context, pyrosequencing analysis revealed increasing spontaneous methylation of the *p16* ^*INK4a*^ promoter in BPECs with PDs. Therefore, while absence of p16 protein expression at the pre-stasis PDs probably reflects the low stress conditions in which BPECs are grown, lack of p16 protein at mid and late PDs (post-stasis) most probably results from inactivation of the gene encoding this tumor suppressor protein. Silencing of the gene encoding p16^INK4a^ is a common event during the transformation process of a variety of human epithelial cells [27–30]. Certainly, there is now substantial evidence that multiple tumor suppressor genes are increasingly methylated with age (reviewed in [31]). Specifically, CpG islands from the promoter region of *p16* ^*INK4a*^ become methylated during aging in normal tissues, likely favoring malignant transformation [32]. Thus, far from precluding the use of this cell model, our results suggest that, in contrast to other cell types, breast epithelial cells have a particular characteristic that might contribute to their malignant transformation: they frequently and spontaneously evade cell cycle checkpoints with ensuing chromosome instability.

Suppression of the checkpoint controller p16^INK4a^ allows BPECs to escape a senescence-like barrier that they encounter when grown in vitro. As shown here, propagation of these cells, which have undetectable hTERT, leads to telomere uncapping and end-to-end fusion of uncapped chromosomes. The formation of unbalanced chromosome rearrangements, aneuploid chromosome segregation and tetraploidization clearly indicates that these cells develop high levels of chromosome instability. According to a telomere-dependent origin of this chromosome instability, we observed that the distribution of chromosomes involved in structural aberrations correlates with the profiles of telomere signal-free ends. To further support these results, we observed that restoration of telomerase activity by expression of ectopic *hTERT* rescues the chromosomal instability phenotype of BPECs. Most importantly, the genomic alterations observed in BPECs closely resemble those seen in human breast cancers, which like most other human carcinomas harbor gross unbalanced structural and numerical chromosome aberrations.

## Conclusions

The contribution of telomere dysfunction in human breast carcinogenesis is a general notion that has been progressively tuned. The strongest evidence that supports this idea arises from a whole-genome analysis of cancer cells to detect chromosome end-to-end fusions by a PCR-based assay [33]. This study provided direct evidence that human breast lesions, but not normal breast tissues, contained telomere fusions. Importantly, in normal breast tissue, the luminal cells in histologically normal terminal ductal lobular units have shorter telomeres than myoepithelial cells [34–36]. This may result from differences in the cell cycle. Luminal cells proliferate periodically with the menstrual cycle, while mature myoepithelial cells are essentially non-proliferative. In line with these *in situ* studies, here we report that breast primary epithelial cells derived from normal mammary gland explants develop telomere dysfunction and rampant chromosome instability when propagated in vitro. Altogether, our results suggest that telomere dysfunction triggers the genomic instability necessary for breast carcinoma initiation, and provide cells with the constellation of genomic changes needed for malignant transformation.

## Additional files

Additional file 1: Table S1. Primers used for qRT-PCR. (PDF 70 kb
Additional file 2: Figure S1. Methylation mean of the *p16* ^*INK4a*^ promoter gene in 14BPECs at different population doublings, as obtained by pyrosequencing methylation analyses. *BPECs* breast primary epithelial cells. (PDF 68 kb)
Additional file 3: Figure S2. Chromosome instability in 14BPEC. **a** Abnormal nuclear morphologies. Frequency of abnormal nuclear morphologies per binucleated cell in 14BPECs at initial, medium and late population doubling (PD). **b** Frequencies of the different types of structural chromosome aberrations observed in metaphase plates of 14BPEC at an early, a mid and a late PD. **c** Frequencies of aneuploidy in 14BPECs at an early, a mid and a late population doubling. **d** Frequencies of tetraploidy in 14BPECs at an early, a mid and a late population doubling. *BPECs* breast primary epithelial cells. (PDF 101 kb)
Additional file 4: Figure S3. Telomere attrition in finite breast primary epithelial cells (BPECs). **a** Frequencies of telomere signal-free ends in metaphase plates of 12BPEC at an early, a mid and a late population doubling. **b** Frequencies of telomere signal-free ends in metaphase plates of 14BPEC at an early, a mid and a late population doubling. (PDF 91 kb)
Additional file 5: Figure S4. Tetraploidy. Frequencies of tetraploid 14BPEC at metaphase (endo-reduplicated and non-endo-reduplicated) at the three population doublings (PD) analyzed. *BPECs* breast primary epithelial cells. (PDF 89 kb)
Additional file 6: Figure S5. mRNA levels of human telomerase reverse transcriptase (hTERT) assessed by qRT-PCR in 04BPEC-hTERT (**a**) and 05BPEC-hTERT (**b**) versus finite breast primary epithelial cells (BPECs) (non-transduced). (PDF 68 kb)
Additional file 7: Table S2. Chromosome analysis of 04BPEC-hTERT and 05BPEC-hTERT. (PDF 70 kb)

## Abbreviations

ANM: abnormal nuclear morphologies
BPEC: breast primary epithelial cell
BSA: bovine serum albumin
CIN: chromosome instability
DAPI: 4',6-diamidino-2-phenylindole
FISH: fluorescence in situ hybridization
FITC: fluorescein isothiocyanate
GAPDH: glyceraldehyde-3-phosphate dehydrogenase
HMEC: human mammary epithelial cell
hTERT: human telomerase reverse transcriptase
MEGM: mammary epithelial growth medium
MEpiCM: mammary epithelial growth medium-basal
p16^IKN4a^: cyclin-dependent kinase inhibitor 2A
p53: cellular tumor antigen p53 protein
PBS: phosphate-buffered saline
PD: population doubling
PNA: peptide nucleic acid
SA-β Gal: senescence-associated β-galactosidase
stasis: stress or aberrant signaling-induced senescence
