# Supplementary material for: Breast primary epithelial cells that escape p16-dependent stasis enter a telomere-driven crisis state
Source: Breast Cancer Res. 2016 Jan 13;18:7. doi: 10.1186/s13058-015-0667-z (PMC4711177; doi:10.1186/s13058-015-0667-z)
Supplement: Additional file 1: Table S1. — Primers used for qRT-PCR. (PDF 70 kb [file 13058_2015_667_MOESM1_ESM.pdf]

Gene	Use	Oligonucleotide sequence (5'->3')
GAPDH	FW	AGCCACATCGCTCAGACAC
	RV	GCCCAATACGACCAAATCC
p53	FW	CCTCACCATCATCACACTGG
	RV	TTGCGGAGATTCTCTTCCTC
TERT	FW	CTCCATCCTGAAAGCCAAGAA
	RV	AGTCAGCTTGAGCAGGAATG
p16^{INK4a}	FW	CCCAACGCACCGAATAGTTA
	RV	CACCAGCGTGTCCAGGAA
KRT19 (K19)[a]	FW	GCACTACAGCCACTACTACACGA
	RV	CTCATGCGCAGAGCCTGTT

[a](Stathopoulou et al 2003)
