# Supplementary figures and images for: Breast primary epithelial cells that escape p16-dependent stasis enter a telomere-driven crisis state
Source: Breast Cancer Res. 2016 Jan 13;18:7. doi: 10.1186/s13058-015-0667-z (PMC4711177; doi:10.1186/s13058-015-0667-z)

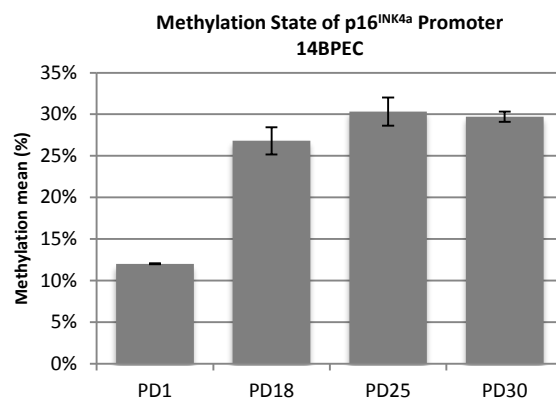

Supplement: Additional file 2: Figure S1. — Methylation mean of the p16 INK4a promoter gene in 14BPECs at different population doublings, as obtained by pyrosequencing methylation analyses. BPECs breast primary epithelial cells. (PDF 68 kb) [file 13058_2015_667_MOESM2_ESM.pdf]

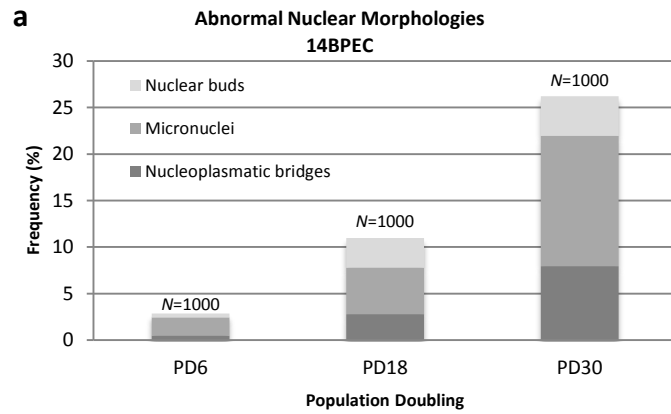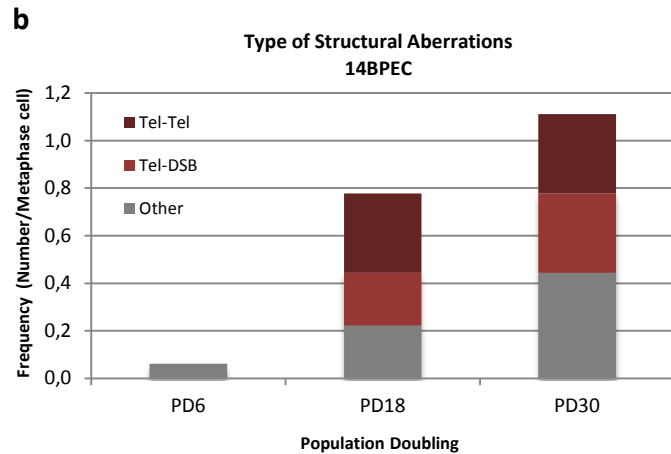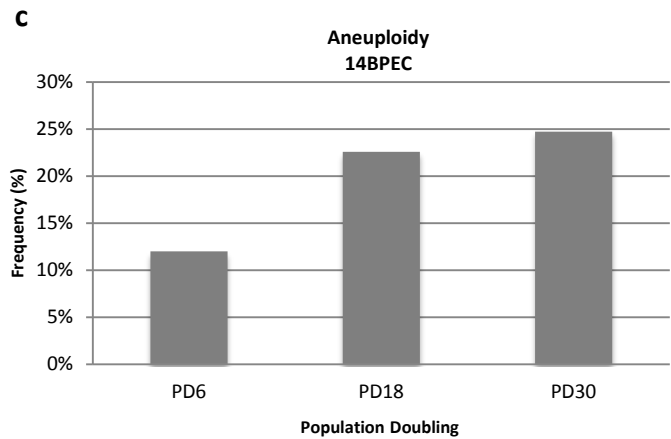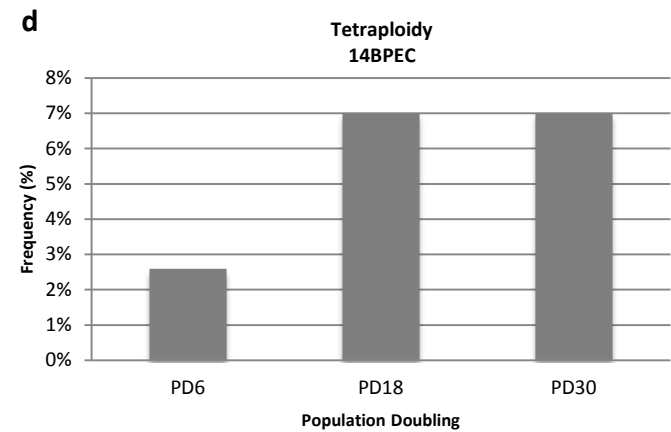

Supplement: Additional file 3: Figure S2. — Chromosome instability in 14BPEC. a Abnormal nuclear morphologies. Frequency of abnormal nuclear morphologies per binucleated cell in 14BPECs at initial, medium and late population doubling (PD). b Frequencies of the different types of structural chromosome aberrations observed in metaphase plates of 14BPEC at an early, a mid and a late PD. c Frequencies of aneuploidy in 14BPECs at an early, a mid and a late population doubling. d Frequencies of tetraploidy in 14BPECs at an early, a mid and a late population doubling. BPECs breast primary epithelial cells. (PDF 101 kb) [file 13058_2015_667_MOESM3_ESM.pdf]

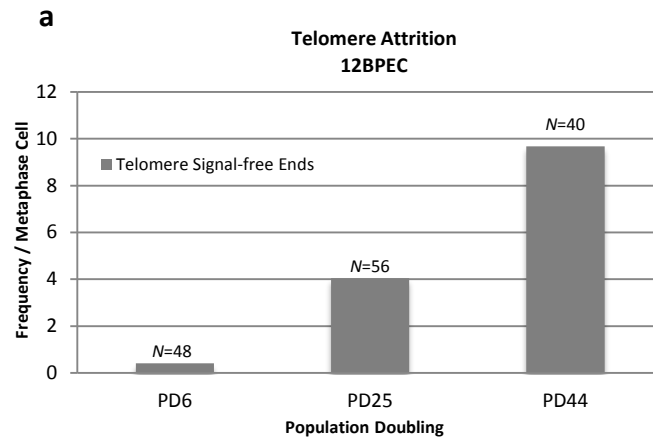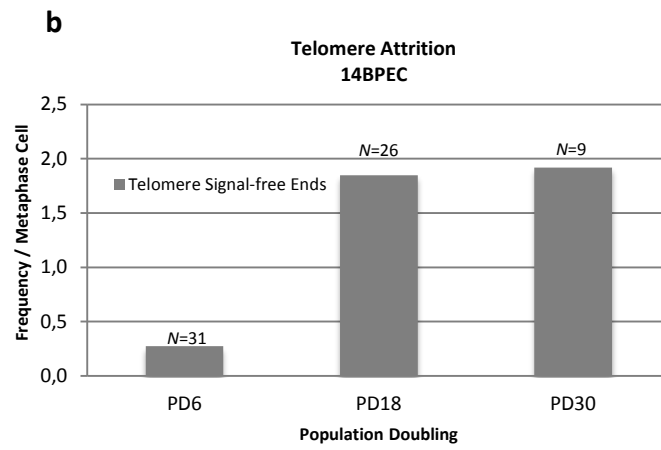

Supplement: Additional file 4: Figure S3. — Telomere attrition in finite breast primary epithelial cells (BPECs). a Frequencies of telomere signal-free ends in metaphase plates of 12BPEC at an early, a mid and a late population doubling. b Frequencies of telomere signal-free ends in metaphase plates of 14BPEC at an early, a mid and a late population doubling. (PDF 91 kb) [file 13058_2015_667_MOESM4_ESM.pdf]

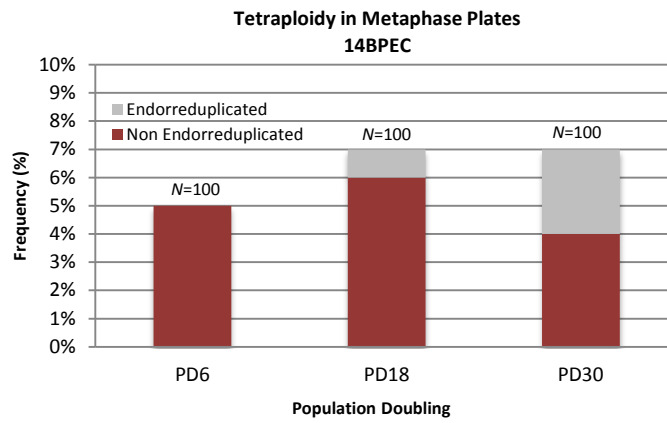

Supplement: Additional file 5: Figure S4. — Tetraploidy. Frequencies of tetraploid 14BPEC at metaphase (endo-reduplicated and non-endo-reduplicated) at the three population doublings (PD) analyzed. BPECs breast primary epithelial cells. (PDF 89 kb) [file 13058_2015_667_MOESM5_ESM.pdf]

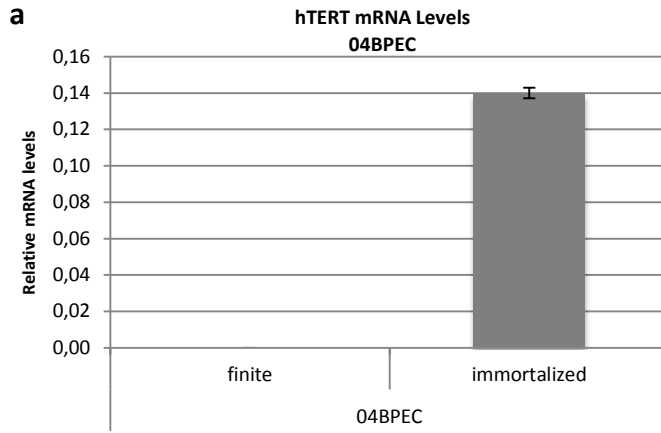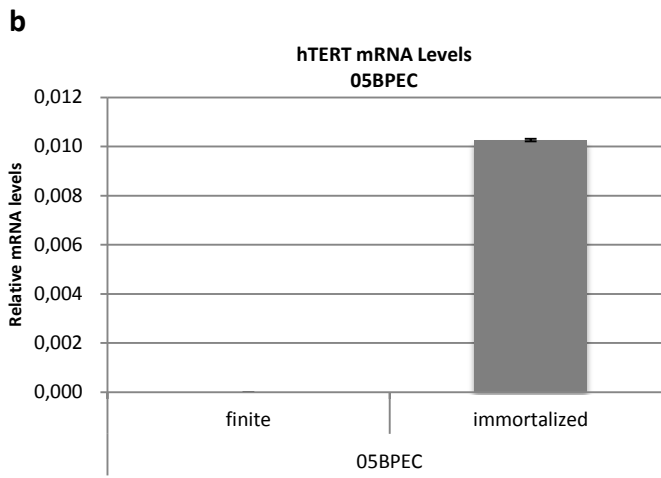

Supplement: Additional file 6: Figure S5. — mRNA levels of human telomerase reverse transcriptase (hTERT) assessed by qRT-PCR in 04BPEC-hTERT (a) and 05BPEC-hTERT (b) versus finite breast primary epithelial cells (BPECs) (non-transduced). (PDF 68 kb) [file 13058_2015_667_MOESM6_ESM.pdf]
