# Supplementary material for: Breast primary epithelial cells that escape p16-dependent stasis enter a telomere-driven crisis state
Source: Breast Cancer Res. 2016 Jan 13;18:7. doi: 10.1186/s13058-015-0667-z (PMC4711177; doi:10.1186/s13058-015-0667-z)
Supplement: Additional file 7: Table S2. — Chromosome analysis of 04BPEC-hTERT and 05BPEC-hTERT. (PDF 70 kb) [file 13058_2015_667_MOESM7_ESM.pdf]

Cell	PD	N	N of Telomere Signal-free Ends	Chromosome Aberration[a]	Tetraploidy[b]
**04BPEC**	18	16	62	0,188	0,039
**05BPEC**	26	15	23	0,133	0,047
**04BPEC-hTERT**	80	24	0	0,038	0
**05BPEC-hTERT**	90	26	0	0	0

[a] Values indicate frequency of non-clonal chromosome aberration per cell.
[b] Values indicate frequency of tetraploidy per cell
